# Occlusion intestinale sur diverticule de Meckel: à propos d'un cas

**DOI:** 10.11604/pamj.2019.32.117.16523

**Published:** 2019-03-13

**Authors:** Pius Wonga Omole, Didier Tshibangu Mujinga, Nasser Amisi Lubosha, Igor Mujinga Wa Mujinga, Daniel Ilunga Ntanga

**Affiliations:** 1 Département de Chirurgie, Cliniques Universitaires de Lubumbashi, Faculté de Médecine, Université de Lubumbashi,Lubumbashi, République Démocratique du Congo

**Keywords:** Meckel's diverticulum, bowel obstruction, omphalomesenteric channel, Diverticule de Meckel, occlusion intestinale, canal omphalomésenterique

## Abstract

Le diverticule de Meckel est un reliquat du canal omphalomésenterique. Ce diverticule peut se perforer, s'enflammer aussi créer une occlusion. Les auteurs rapportent le cas d'un homme âgé de 30 ans, hospitalisé et pris en charge dans les cliniques universitaires de Lubumbashi pour occlusion intestinale et dont le constat per opératoire était un volvulus du grêle sur diverticule de Meckel avec nécrose intestinale. Son évolution était bonne après l'intervention chirurgicale.

## Abstract (en)

Meckel's diverticulum is a remnant of the omphalomesenteric channel. Diverticulum may result in perforation, inflammation an even in occlusion. We here report the case of a 30-year old man, hospitalized and treated for bowel obstruction at the university clinics of Lubumbashi. Intraoperative findings showed small bowel volvulus due to Meckel's diverticulum associated with intestinal necrosis. Patient's outcome was favorable after surgery.

## Introduction

Durant la période embryonnaire précoce (2^ème^ et 4^ème^ semaine du développement après la conception), l'embryon est constitué de 3 feuillets: mésoblaste, endoblaste et ectoblaste [1]. Tous ces feuillets convergents antérieurement vers l'ombilic de manière concomitante. L'intestin moyen communique avec l'ombilic par le canal vitellin (canal omphalomésenterique) qui disparait à la 10^ème^ semaine de la vie embryonnaire lors de la réintégration des anses dans l'abdomen [1,2]. La persistance partielle de ce canal est appelé Diverticule de Meckel. C'est l'anomalie congénitale la plus fréquente du tractus gastro-intestinale avec une légère prédominance masculine [3,4]. Elle est rare et est rencontrée chez 2 à 4% de la population [3]. Le diverticule de Meckel reste le plus souvent asymptomatique et n'est diagnostiqué que fortuitement ou lors de la survenue des complications telles que: l'hémorragie digestive, l'occlusion intestinale, l'invagination intestinale, la diverticulite de Meckel, la perforation, la fistule ombilicale et la dégénérescence tumorale [3,5-8]. Ces complications sont fréquentes chez l'enfant, d'autant plus qu'il est jeune. Cependant elles ne sont pas habituelles chez l'adulte [9]. L'objectif de cet article était de décrire le cas clinique et la prise en charge de volvulus du grêle sur diverticule de Meckel observé dans les cliniques universitaires de Lubumbashi chez un adulte de 30 ans en juin 2017.

## Patient et observation

**Figure 1:**
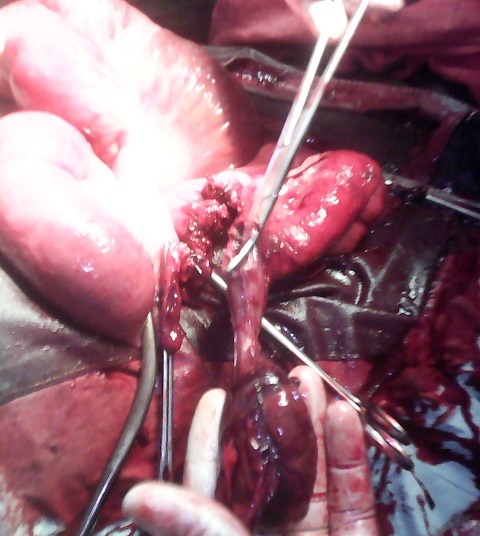
La poche de diverticule de Meckel sectionnée per opératoire

**Figure 2:**
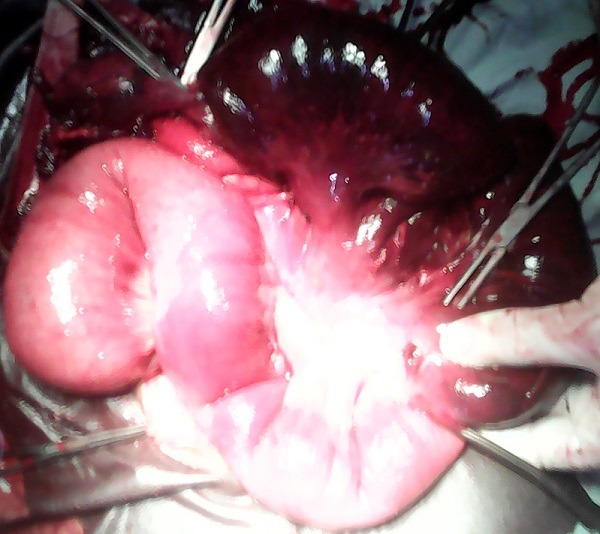
La portion de l'intestin du grêle nécrosé sur ou moins 75cm

Il s'agissait d'un homme âgé de 30 ans transféré d'un centre hospitalier de la place vers le service de médecine interne des Cliniques Universitaires de Lubumbashi (CUL) pour fièvre. Ce transfert a eu lieu après trois jours de traitement sans succès. Au service de médecine interne, une échographie abdominale a été réalisée et avait révélé une dilatation des anses intestinales et un épanchement liquidien abdominal de moyenne abondance. Cette échographie a motivé le transfert du patient vers le service de chirurgie. Ses antécédents étaient non contributifs à sa pathologie. Les plaintes du patient étaient une douleur abdominale permanente avec des paroxysmes, localisée à l'épigastre; vomissement et arrêt des gaz depuis 1 jour. L'examen physique de l'abdomen a révélé: un ballonnement abdominal diffus plus marqué dans la moitié supérieure de l'abdomen; une défense abdominale diffuse; une matité mobilisable dans les deux flancs; et présence des bruits hydro-aériques à l'auscultation. Les orifices inguinaux étaient libres. Le toucher rectal a révélé un cul-de-sac de Douglas non bombant mais sensible. La radiographie de l'abdomen sans préparation n'avait pas été réalisée. Le diagnostic d'occlusion intestinale aigüe a été retenu. Et après le bilan sanguin préopératoire, la visite pré anesthésique et la réanimation préopératoire, une laparotomie exploratrice a été réalisée. L'inventaire des lésions a révélé ce qui suit: 1) un intestin grêle fortement dilaté et un colon ratatiné mais d'aspect normal; 2) un volvulus du grêle à 60cm de valvule de Bauhin. L'intestin a été dévolvulé, et un diverticule de Meckel long de 8cm et 5cm de diamètre a été mis en évidence (Figure 1) sur le bord ante mésentérique. C'est autour de ce diverticule que le grêle avait volvulé. La portion de l'intestin volvulé était sphacélé sur une longueur de 75cm (Figure 2). L'acte opératoire a consisté en une résection de la portion sphacélée emportant le diverticule de Meckel suivie d'une anastomose termino-terminale. Les suites postopératoires ont été simples. Sa sortie a été autorisée au 14^ème^jour. L'examen histo-pathologique du diverticule a montré un tissu fibreux, siège d'une importante réaction inflammatoire, la muqueuse était semblable à celle de la muqueuse gastrique.

## Discussion

Le diverticule de Meckel est la persistance partielle du canal omphalomésenterique. C'est l'anomalie congénitale la plus fréquente du tractus gastro-intestinal avec une légère prédominance masculine [3,4]. Il est rare et rencontré entre 2 à 4% de la population [2,3]. Le diverticule de Meckel reste le plus souvent asymptomatique et n'est diagnostiqué que fortuitement ou lors de la survenue des complications. Cependant elles ne sont pas habituelles chez l'adulte [5-7]. Le diagnostic d'une occlusion intestinale due au diverticule de Meckel peut être évoqué en préopératoire, soit à l'échographie abdominale, à la scintigraphie au technétium 99m, à la tomodensitométrie abdominale ou à l'imagerie par résonnance magnétique(IRM) [7]. L'occlusion mécanique est la complication la plus fréquente chez l'adulte; elle représente 24 à 53%. Le plus souvent il s'agit d'une occlusion avec mécanisme variable [5,6,9]: volvulus, invagination, fixation de diverticule à l'ombilic ou en tout autre point de l'abdomen. La fréquence des complications est légèrement plus importante chez l'homme [3,4]. Dans le cas présenté, il s'agit d'un homme ayant présenté un volvulus du grêle. La localisation du diverticule de Meckel varie entre 10 et 100 cm par rapport à la valvule de Bauhin dans 50% des cas, ses dimensions sont en moyenne 2cm de diamètre, 5cm de longueur [4], les diverticules sont constitués d'une hétérotopie muqueuse, de type gastrique dans 23 à 60% des cas; il peut s'agir d'une muqueuse de type pancréatique. Dans cette étude, le diverticule de Meckel était situé à 60cm de valvule de Bauhin avec 5cm de diamètre et 8cm de long avec une muqueuse de type gastrique. Edgar Ouangré *et al.* ont réalisé une étude de 11 cas de diverticule de Meckel. Au cours de cette étude l'âge moyen était de 29,8 ans, il y avait 8 cas d'occlusion intestinale avec résection segmentaire iléale emportant le diverticule de Meckel avec rétablissement de la continuité digestive. Dans le cas présenté, il y a eu résection intestinale emportant le diverticule de Meckel avec rétablissement de la continuité. Il faut savoir évoquer le diagnostic de diverticule de Meckel au sein du vaste groupe des occlusions intestinales aigues ou subaigües notamment chez le sujet jeune sans antécédents chirurgicaux, car le diverticule de Meckel est difficile à identifier malgré les progrès de l'imagerie en coupe [3]. Il faut savoir le reconnaitre dans le diagnostic des douleurs abdominales aigues afin de guider au mieux la prise en charge chirurgicale.

## Conclusion

Le diverticule de Meckel est la persistance partielle du canal omphalomésenterique, Il est rare et rencontré entre 2 à 4% de la population. Il reste le plus souvent asymptomatique et n'est diagnostiqué que fortuitement ou lors de la survenue des complications. Cependant elles ne sont pas habituelles chez l'adulte. Il faut savoir le reconnaitre dans le diagnostic des douleurs abdominales aigues afin de guider au mieux la prise en charge chirurgicale. L'évolution dépend de la précocité du diagnostic.

## Conflits d'intérêts

Les auteurs ne déclarent aucun conflit d'intérêts.
